# Bioprinting of GelMA-Based Hydrogels to Aid in Creation of Biomimetic 3D Models for Glioblastoma

**DOI:** 10.3390/mi16060654

**Published:** 2025-05-29

**Authors:** Kaitlyn Ann Rose Schroyer, Kylie Marie Schmitz, Gunjeeta Raheja, Bin Su, Justin D. Lathia, Liqun Ning

**Affiliations:** 1 Department of Chemical and Biomedical Engineering, Cleveland State University, Cleveland, OH 44115, USA; k.schroyer@vikes.csuohio.edu (K.A.R.S.);; 2 Department of Mechanical Engineering, Cleveland State University, Cleveland, OH 44115, USA; g.raheja@vikes.csuohio.edu; 3 Center for Gene Regulation in Health and Disease, Department of Chemistry, Cleveland State University, Cleveland, OH 44115, USA; b.su@csuohio.edu; 4 Case Comprehensive Cancer Center, Cleveland, OH 44106, USA; lathiaj@ccf.org; 5 Cleveland Clinic, Cleveland, OH 44195, USA

**Keywords:** glioblastoma, tumor microenvironment, extracellular matrix, 3D bioprinting, hydrogels, GBM modeling

## Abstract

Glioblastoma (GBM, isocitrate dehydrogenase wild-type) is the most common primary malignant brain tumor in adults and is associated with a severely low survival rate. Treatments offer mere palliation and are ineffective, due, in part, to a lack of understanding of the intricate mechanisms underlying the disease, including the contribution of the tumor microenvironment (TME). Current GBM models continue to face challenges as they lack the critical components and properties required. To address this limitation, we developed innovative and practical three-dimensional (3D) GBM models with structural and mechanical biomimicry and tunability. These models allowed for more accurate emulation of the extracellular matrix (ECM) and vasculature characteristics of the native GBM TME. Additionally, 3D bioprinting was utilized to integrate these complexities, employing a hydrogel composite to mimic the native environment that is known to contribute to tumor cell growth. First, we examined the changes in physical properties that resulted from adjoining hydrogels at diverse concentrations using Fourier-Transform Infrared Spectroscopy (FTIR), compression testing, scanning electron microscopy (SEM), rheological analysis, and degradation analysis. Subsequently, we refined and optimized the embedded bioprinting processes. The resulting 3D GBM models were structurally reliable and reproducible, featuring integrated inner channels and possessing tunable properties to emulate the characteristics of the GBM ECM. Biocompatibility testing was performed via live/dead and AlamarBlue analyses using GBM cells (both commercial cell lines and patient-derived cell lines) encapsulated in the constructs, along with immunohistochemistry staining to understand how ECM properties altered the functions of GBM cells. The observed behavior of GBM cells indicated greater functionality in softer matrices, while the incorporation of hyaluronic acid (HA) into the gelatin methacryloyl (gelMA) matrix enhanced its biomimicry of the native GBM TME. The findings underscore the critical role of TME components, particularly ECM properties, in influencing GBM survival, proliferation, and molecular expression, laying the groundwork for further mechanistic studies. Additionally, the outcomes validate the potential of leveraging 3D bioprinting for GBM modeling, providing a fully controllable environment to explore specific pathways and therapeutic targets that are challenging to study in conventional model systems.

## 1. Introduction

Glioblastoma (GBM, isocitrate dehydrogenase wild-type) is the most common primary malignant brain tumor in adults [1,2]. Despite advancements in comprehensive treatments, including maximal safe surgical resection, chemotherapy, and radiotherapy, the five-year survival rate remains alarmingly low [3,4]. This persistent challenge is primarily attributed to the inherent complexity of GBM physiology, which hampers our understanding of its underlying mechanisms and delays the development of effective therapies. It has been documented that GBM comprises not only cancer cells but also a diverse array of other biological components, including stromal cells, the extracellular matrix (ECM), vascular structures, and a variety of complex molecules, collectively forming a unique tumor microenvironment (TME) [5,6,7]. Among these components, the interactions between GBM cells and their TME are widely recognized as critical determinants of tumor behavior [8,9,10]. These interactions determine many cancer hallmarks, such as enhanced cell proliferation, resistance to cell death, and therapeutic resistance, highlighting the intricate and reciprocal relationship between GBM cells and their surrounding microenvironment [11,12,13,14,15,16]. Specifically, mechanical properties of the ECM have been demonstrated to critically affect GBM cell phenotypes, as GBM development is accompanied by changes in ECM stiffness and composition due to the increased deposition of ECM materials such as collagen and hyaluronic acid (HA) [5,7,17,18,19,20,21]. The elevated mechanical stiffness of the ECM and alterations in ECM components may be closely related to glioma grade, affecting the survival and proliferation of GBM cells [22,23,24]. Thus, generating platforms with the ability to recapitulate the inherent connections between ECM modifications and GBM cell functions could offer significant insights for GBM mechanism exploration.

A variety of GBM models have been developed with the aim of replicating the multifaceted characteristics of human GBM or, at the very least, capturing certain key features critical to its pathological characteristics [7,25,26]. When efforts are directed toward elucidating the role of the ECM, it is essential that the employed models accurately replicate the key features of the ECM to ensure relevant insights. The most widely used GBM models, including monolayer cell cultures and animal models, face challenges of dimensionality constraints and species differences, with limited ability to recreate a GBM environment that involves a fully tunable ECM [27,28,29,30]. In response, 3D GBM alternatives, including GBM cell spheroid/organoid models, microfluidic chip models [31], and 3D bioprinted models have been developed [32,33]. GBM cell spheroids/organoids, formed by a self-assembly process of cells, offer improved cellular heterogeneity using single or multiple cell types [34]; however, this approach normally lacks the control and representation of the tumor ECM [33,35]. Microfluidic chips can emulate GBM components through the provision of vascular-like structures and dynamic flow [36,37]. They have demonstrated success in replicating cancer and stromal cells in vitro, but they commonly use substances that create inflexible cell–substrate interfaces on small scales, preventing the reproduction of necessary mechanical and compositional complexity [38,39,40,41,42]. Advancements in 3D bioprinting, particularly its ability to precisely integrate multiple cell types within ECM-derived hydrogels, position it as a powerful platform for constructing in vitro GBM models. This technology allows for the accurate deposition of cells and biomaterials into predefined, biomimetic architectures, effectively overcoming the limitations of monolayer cultures, including limited heterogeneity and restricted dimensionality [32,43,44,45,46,47,48,49,50,51,52,53,54,55,56]. Among the various bioprinting techniques, extrusion-based bioprinting stands out for its ability to create complex geometries by accommodating a wide range of hydrogels, such as collagen, HA, and gelatin. This method employs a delicate fabrication process, preserving the viability and functionality of cells encapsulated within ECM-derived biomaterials [57,58]. Such cellular encapsulation effectively replicates the dynamic interactions between cells and the ECM observed in natural tissues. However, conventional hydrogel-based bioprinted disease models often face significant challenges, including low structural fidelity and mechanical instability, which can lead to structural failure [59,60]. These limitations are primarily due to the restricted printability of hydrogels. It is critical to overcome these limitations for the successful fabrication of complex models, such as GBM models incorporating vascular structures. By developing these complex structures, models will more accurately represent vascular components that are vital to GBM tumor growth and resistance against chemotherapy.

The development of embedded bioprinting presents a promising solution to the challenges associated with low structural fidelity and instability in conventional bioprinting [58,61,62]. This technique utilizes a viscoelastic supporting bath with a defined yield stress to stabilize extruded filaments during the bioprinting process. The external support provided by the bath ensures the structural integrity of the printed constructs, enabling the use of soft and mechanically weak hydrogels without the risk of collapse. However, embedded bioprinting necessitates careful optimization, as the flow behavior of hydrogel inks can vary significantly, requiring precise calibration of the bioprinting parameters. We previously refined the use of a Carbopol-based bath for embedded bioprinting and successfully developed strategies to print gelMA, a widely utilized hydrogel derived from the partial hydrolysis of collagen [57,58]. In the context of GBM, collagen provides a structurally supporting invasive network for GBM cells, while HA is a primary ECM constituent in healthy brain tissues and is excessively expressed in GBM. HA interacts with cell surface receptors, activating intracellular signaling pathways that drive GBM cell migration and invasion [63,64]. Thus, developing gelMA-HA bioinks with precisely controlled HA content is particularly appealing for the construction of GBM models. These models not only enable the study of mechanical behavior (dominated by gelMA) but also provide insights into the effects of ECM components (dominated by HA) on GBM progression. Therefore, it is crucial to optimize the embedded bioprinting process for gelMA-HA bioinks.

The goal of this study was to develop an embedded bioprinting process for creating gelMA-HA-based, vessel channel-integrated GBM models. These models were designed with tunable mechanical properties and biological components, enabling the investigation of how variations in these properties influence GBM cell behavior. Initially, we conducted a comprehensive assessment of the formulation of gelMA-HA inks, focusing on their chemical and mechanical properties. We then optimized the bioprinting parameters, including temperature, pressure, and speed, based on the rheological characteristics of the inks. This method facilitated the development of a robust bioprinting process capable of fabricating hollow channel-integrated 3D GBM models with high structural fidelity and reproducibility. Subsequently, we examined the effects of ECM mechanical properties and HA concentration on GBM cell viability, proliferation, and protein expression. Specifically, we focused on the expression of androgen receptor (AR) and heat shock protein 27 (HSP27) through immunostaining, as the AR–HSP27 axis has emerged as a novel therapeutic target for GBM, supported by evidence of sex-based differences in GBM progression. The findings of this study will have a broad and immediate impact on the field as they establish a biomanufacturing method for fabricating cell-encapsulated, hydrogel-based 3D GBM models with high structural accuracy. These models feature customizable properties, providing a reliable in vitro platform to investigate the critical TME factors influencing GBM cell function. By incorporating additional factors known to affect GBM progression, this model offers a much-needed alternative to existing models, serving as a platform for further GBM research.

## 2. Materials and Methods

### 2.1. Materials

GelMA synthesis materials were procured from Sigma (St. Louis, MO, USA), including gelatin from porcine skin (G2500) and methacrylic anhydride (276685). HA (53747), Irgacure (410896), and culture media (Dulbecco’s Modified Eagle’s Medium and Fetal Bovine Serum) were purchased from Sigma as well. Carbopol ETD 2020 polymer was sourced from Lubrizol (Wickliffe, OH, USA). Live/Dead, including calcein-AM and ethidium homodimer, and AlamarBlue assay kits were obtained from Biotium (Fremont, CA, USA). Antibodies, including primary HSP27 and D6F11 (for androgen receptor, AR) antibodies and Alexa Fluor 488 (Goat anti-Rabbit IgG (H + L) Cross-Adsorbed Secondary Antibody) and 555 (Goat anti-Mouse IgG (H + L) Cross-Adsorbed Secondary Antibody), were purchased from ThemoFisher (Waltham, MA, USA). The GBM cell line (U87) was provided by our collaborator, Dr. Bin Su, from the Department of Chemistry at Cleveland State University (Cleveland, OH, USA). The DI318 human GBM cell line and culture medium were provided by Dr. Justin Lathia from the Cleveland Clinic (Cleveland, OH, USA).

### 2.2. GelMA Synthesis and GelMA-HA Preparation

GelMA was synthesized in-house following our developed protocol [65]. Briefly, 10 g of gelatin was added to a flask heated to 50 °C with 100 mL of 1X PBS (Corning, Corning, NY, USA) and agitated for 30 min. Methacrylic anhydride was then introduced dropwise while continuously stirring. After 3 h, the solution was centrifuged, and the supernatant was returned to the flask with an additional 150 mL of preheated PBS (50 °C) and agitated for another 15 min to terminate the reaction. The solution was dialyzed in distilled water for 7 days with twice-daily water changes and then sterile-filtered (0.22 μm, Corning), frozen overnight, and lyophilized (Labconco, Kansas City, MO, USA) for 5 days. The dried material was resuspended in 0.5% *w*/*v* Irgacure in PBS, producing final gelMA concentrations of 4% and 10% *w*/*v*. For gelMA-HA composites, HA was mixed with Irgacure prior to freeze-dried gelMA addition and dissolved at 37 °C for at least 3 h to reach final HA concentrations of 0.1%, 0.2%, 0.5%, and 1% *w*/*v*. HA that was sterilized under UV light for 1 h was used for cell-incorporated applications.

### 2.3. Carbopol Solution Preparation

Carbopol powder was gradually incorporated into deionized water to reach a concentration of 0.5% *w*/*v* and then stirred at 1000 rpm at room temperature (23 °C) overnight to ensure thorough dispersion. The suspension was aliquoted into 50 mL centrifuge tubes and diluted with deionized water to obtain a final Carbopol medium concentration of 0.4% *w*/*v*. The pH was later adjusted by adding 4 M NaOH in measured amounts to achieve a balanced medium. The pH-adjusted Carbopol medium was centrifuged at 3500 rpm for 10 min to remove air bubbles and then stored at 4 °C for subsequent use. For cell printing, Carbopol powder was first sterilized via 40 min of UV exposure and then dissolved following the same procedure under sterile conditions to ensure an aseptic environment.

### 2.4. Hydrogel Composite Characterization Using Fourier-Transform Infrared Spectroscopy (FTIR)

To analyze the structural characteristics of hydrogel composites, pure gelMA and gelMA-HA samples were prepared by pipetting bioink droplets with varying HA concentrations onto culture dishes. The droplets were cured and examined using FTIR with the Perkin-Elmer FTIR microscope accessory, Spectrum Two. Spectral data were collected over a range of 3950 to 550 cm^−1^ at a resolution of 2 cm^−1^, providing insights into the molecular composition and interaction between gelMA and HA components within the hydrogel matrix.

### 2.5. Mechanical Evaluation

The mechanical stiffness of hydrogel samples was measured by uniaxial compression on a CellScale tester installed with a 10 N load cell. Cylindrical hydrogels (4 mm height, 10 mm diameter) from casting were prepared and compressed at 0.025 mm/s to 50% of the initial height. Young’s moduli were derived from the stress–strain curves at the linear region, with three replicates per group for statistical analysis.

### 2.6. Scanning Electron Microscopy (SEM)

SEM was employed to characterize cured porous structures of gelMA and gelMA-HA hydrogels. For this, samples were molded, cured (10 mW/cm^2^ UV light with an exposure time of 30 s on each side of the avascular model), frozen, and lyophilized before imaging with an Inspect F50 Field Emission SEM. Pore size, distribution, and porosity were analyzed using ImageJ software (version 1.54m) from four randomly selected images per sample.

### 2.7. Degradation Analysis

Degradation studies were performed on acellular hydrogel samples composed of 10% GelMA, as well as on 10% GelMA samples supplemented with varying concentrations of HA, ranging from 0.1% to 1%. The samples were incubated in PBS at 37 °C for 7 days, with the media refreshed daily. The initial weight of each sample was recorded on day 0, followed by daily measurements using a digital scale with a resolution of 0.1 mg. Each material group was prepared in triplicate to ensure reproducibility.

### 2.8. Rheological Temperature Sweep

To determine the optimal viscoelastic range for hydrogel bioinks, we conducted an analysis on uncured 10% *w*/*v* gelMA using an Anton Paar Physical MCR 301 rotational rheometer. A parallel plate geometry with a 25 mm diameter was utilized, maintaining a gap of less than 1 mm to prevent wall slip. Temperature control was achieved with a Peltier device hood (H-PTD200, Anton Paar, Graz, Austria), effectively minimizing sample evaporation throughout testing. A temperature sweep from 4 °C to 40 °C was performed at a rate of 2 °C/min to observe the temperature-dependent behavior of storage and loss moduli in gelMA. The crossover point between the storage and loss moduli was identified, representing the phase-changing temperature of gelMA. This parameter is critical for defining the viscoelastic constraints needed for reliable and consistent bioprinting applications.

### 2.9. The 3D Bioprinting Setup and Parameter Control Process for Two-Layer Design Manufacturing

A two-layer lattice model was utilized for bioprinting parameter optimization. For this model, cylindrical filaments were designed with a 200 μm diameter, matching the specifications of the bioprinting nozzle (27 Gauge, 0.5″ needle length, Cellink, San Diego, CA, USA). The spacing between adjacent filaments was set to 1.8 mm, with the first and second layers oriented at a 90° angle to each other. To initiate printing, gelMA and gelMA-HA hydrogel inks were loaded into syringes for the BioX printer (Cellink). The bioinks were printed under controlled pressure and temperature conditions, maintaining a constant bioprinting speed of 5 mm/s to ensure a stable flow rate and a filament diameter consistent with the nozzle specifications. Trial-and-error experiments were conducted for printing parameter optimization, aiming to produce uniform filaments and maintain the high structural fidelity of the two-layer model.

### 2.10. Printing Fidelity Characterization

To evaluate the fidelity of the bioprinting process, optical microscopy images were captured and analyzed using ImageJ. Three metrics were quantified, including filament diameter ratio, layer angle ratio, and interstrand area ratio [66]. Filament diameter ratio was calculated as the actual printed filament diameter divided by the intended filament diameter, indicating radial deformation. Ratios close to 1 suggested high fidelity, while values deviating significantly from 1 indicated strand distortion. The layer angle ratio measured the alignment between two consecutive printed layers. Ratios near 1 denoted minimal misalignment or distortion, ensuring high fidelity in layer placement. Interstrand area ratio was defined as the area of quadrilateral openings formed by four filaments from adjacent layers; this parameter reflected both strand uniformity and angle precision. Ratios close to 1 indicated high printing fidelity, while significant deviations suggested compromised strand and angle accuracy. Three randomly selected bright-field microscopy images were obtained from each construct for manual measurement and analysis.

### 2.11. Embedded Bioprinting of Inner Channel-Integrated 3D GBM Model

A 3D GBM model featuring an inner ring-shaped channel was designed and bioprinted under optimized conditions within a Carbopol bath to ensure maximum structural fidelity. The design followed the idea of providing a controlled spatial zone of GBM cells that mimicked the heterogeneity in humans while ensuring sufficient cell–vascular interactions in vitro. Following printing, the structures were cross-linked using UV light (10 mW/cm^2^ for 30 s on each side of the printed model) in a Uvitron Intelli-Ray 200 UV box (UVITRON, West Springfield, MA, USA). After crosslinking, the bioprinted models were carefully retrieved from the Carbopol bath and washed in PBS three times on a rocker covered in foil at room temperature (23 °C) to remove any residual Carbopol. To facilitate imaging, the structures were stained with food dye, and dimensional features of the bioprinted model were measured for structural quantification, assessing the precision and integrity of the inner channel design.

### 2.12. Viability of Cells Encapsulated in Hydrogels Using Live/Dead Assay

The viability of U87 and patient-derived DI318 cells encapsulated within gelMA and HA hydrogels was assessed on days 1, 4, and 7. To differentiate living from dead cells, fluorescent dyes, including calcein-AM (1 μg/mL) for live cells and ethidium homodimer (0.5 μg/mL) for dead cells, were added to the cell culture medium. Following 40 min of incubation, the samples were washed with fresh culture medium and observed using an Olympus fluorescence microscope. At each timepoint, three random images were captured and analyzed with ImageJ software. Cell viability was determined by calculating the ratio of live cells to the total cell count.

### 2.13. Cell Proliferation Analysis

Cell proliferation, including both U87 and DI318, was evaluated using the AlamarBlue assay. The AlamarBlue reagent was mixed with fresh cell culture medium at a 1:9 volumetric ratio and added to wells containing cell-encapsulated hydrogels in culture plates. The samples were then incubated for 5 h to allow for dye reduction. After incubation, 100 μL of the medium was collected from each well and transferred to a 96-well plate; then, the absorbance was measured at 550 nm and 600 nm using a microplate reader (BioTek Instruments, Winooski, VT, USA). Measurements were taken on days 1, 4, and 7 to track cell proliferation over time, with four replicates prepared for each time point to ensure statistical reliability.

### 2.14. Immunohistochemistry (IHC) Staining

IHC staining was performed following a protocol established in our laboratory. Briefly, after rinsing with PBS, cell-laden hydrogel samples were fixed in 10% formalin for 30 min, followed by permeabilization with 0.5% *v*/*v* Triton X-100 (Sigma) for 30 min. Samples were then blocked with 3% *w*/*v* bovine serum albumin (BSA, Sigma) for 1 h at room temperature (23 °C). Primary antibodies against HSP27 and D6F11 (1:200 dilution each) were applied and incubated overnight at 4 °C. Following PBS washes, secondary antibodies conjugated with Alexa Fluor 488 and 555 (1:200 dilution each) were added and incubated overnight at 4 °C in the dark. After final PBS rinses, ProLong™ Gold Antifade Reagent with DAPI (Invitrogen, Waltham, MA, USA) was applied for nuclear staining. Imaging was performed using a Nikon Eclipse Ti confocal microscope (Nikon, Melville, NY, USA).

### 2.15. Statistical Analysis

Experimental data were calculated and presented as mean values ± standard deviations (SDs). For each experiment, three to five sample replicates were analyzed (as specified in each section). Statistical significance was assessed using one-way or two-way analysis of variance (ANOVA), with multiple comparisons conducted in GraphPad Prism (version 10.4.1). A significance level of *p* < 0.05 was considered statistically different.

## 3. Results

**Figure 1:**
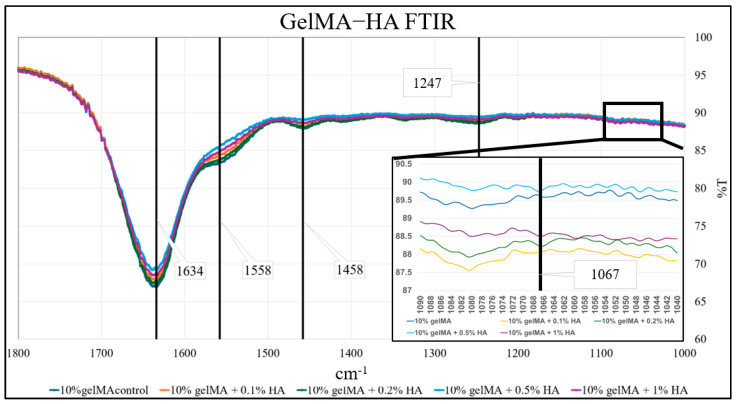
FTIR spectra for 10% gelMA (dark blue), 10% gelMA + 0.1% HA (orange), 10% gelMA + 0.2% HA (green), 10% gelMA + 0.5% HA (light blue), and 10% gelMA + 1% HA (purple), with critical peaks labeled.

Hydrogels have been widely used for modeling human cancers such as GBM tumors in vitro as they enable the construction of soft 3D environments that partially replicate the ECM found within the TME [67]. In vitro modeling allows for absolute control of the model environment, composition, and testing, which is beneficial in shortening analysis times and obtaining the clearest results. In particular, 2D cultures have the advantage of being uncomplicated and inexpensive, making them ideal candidates for basic, preliminary testing. However, this misses the heterogeneity of natural GBM and does not allow for an analysis of interactions between tumor cells and their environment, an important component to include when studying the mechanisms of GBM. In the human GBM, collagen and HA are the two ECM components, offering structural integrity, mechanical support, and a platform for cell signaling and function [68]. Incorporating collagen and HA into GBM models is therefore a rational approach to capturing the essential features. We developed gelMA-HA composites to emulate the composition and mechanical characteristics of native GBM tissue. GelMA is derived from gelatin, which itself is obtained through the partial hydrolysis of collagen [69]. By introducing methacryloyl groups, gelMA gains the capacity to be polymerized under UV or visible light, contingent on the chosen photoinitiators, while preserving the biological properties of gelatin. This modification enables gelMA to act as a versatile ECM substitute that shares amino acid composition, biocompatibility, and biodegradability with collagen, while being able to form stable 3D structures with tunable mechanical strength [70]. We refined a protocol to synthesize gelMA with a controlled degree of functionalization at approximately 60% [58]. This level maintains the natural cell-binding sites of gelMA, preserving the overall biocompatibility of the hydrogel post-crosslinking [71]. In creating the gelMA-HA composite, we physically blended gelMA and HA at precisely controlled concentrations. Upon gelMA crosslinking, the unmodified HA molecules became encapsulated within the formed gelMA polymer network. FTIR data confirmed this integration by revealing that changes in bond structures occurred with the addition of HA (Figure 1). For all groups, the amide I peak was observed at 1634 cm^−1^, amide II was seen at 1558 cm^−1^, and amide III was seen at 1247 cm^−1^, all of which represent the typical groups expressed in gelMA [72,73]. Also, the peak at 1458 cm^−1^ indicated C-H bending vibrations in the methyl group in gelMA. The unique peaks identified at 1067 cm^−1^ for gelMA-HA groups (at different concentrations) indicated the stretching of C-O-C, which corresponded to glycosidic linkages in HA [74]. Also, changes in peak intensities were evident, suggesting that some interactions were present between gelMA and HA molecules. However, these interactions were minimal, which can be attributed to the use of lower HA concentrations. The results confirm the successful incorporation of HA into the gelMA-HA hydrogel following gelMA crosslinking. The inconsistencies in the spectra were likely the result of weak hydrogen bonds between gelMA and HA as HA imparted only a slight increase in the rigidity of the gelMA matrix [68]. A review of the literature reveals that numerous researchers have investigated the incorporation of gelMA with HA, consistently finding that a gelMA-HA mixture is often extremely heterogeneous [75]. The methacrylation of HA (HAMA) has been shown to chemically modify the material, increasing strength and corrosion resistance when compared to pure HA [75]. Since HAMA was not utilized, the gels in this study may have exhibited reduced strength compared to what could be achieved, limiting the material’s relevance. This underscores the presence of irregularities and mechanical complications in the hydrogel, highlighting the need for further synthesis optimization to fabricate a more uniform mixture in future experiments.

**Figure 2:**
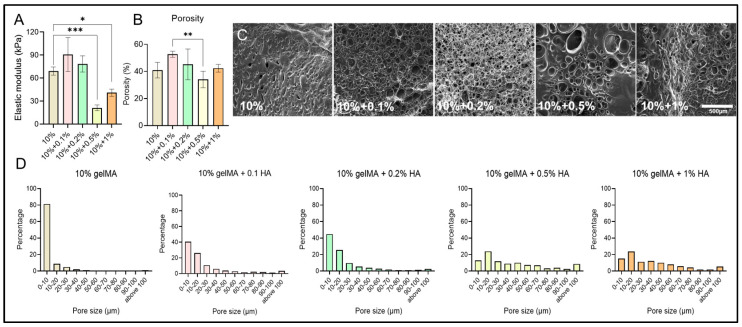
Characterization of gelMA-HA hydrogel substrate properties. (**A**) Elastic moduli of hydrogel substrates with varying HA concentrations. *, **, *** indicates *p* < 0.05 to *p* < 0.001. (**B**) Porosity measurements of hydrogels derived from SEM image analysis. (**C**) SEM images illustrating the microstructures of gelMA-HA hydrogels at different HA concentrations. (**D**) Quantification of micropore size distributions across the hydrogel samples.

We then conducted mechanical tests to quantify the changes in the mechanical stiffness of the hydrogels when HA concentrations were altered. Mechanical stiffness was quantified using the elastic modulus. For pure gelMA, given a constant exposure time of 30 s and UV intensity of 10 mW/cm^2^, the elastic modulus reached 66.27 ± 5.33 kPa (Figure 2A). Interestingly, the addition of HA first increased the elasticity of the hydrogel substrate and then decreased it when the concentration of HA was above 0.2% *w*/*v*. This observation can be explained by the interactions between HA and gelMA [76]. HA is a well-known hydrophilic, viscous hydrogel polymer that is compatible with cellular interactions. The increased viscosity of gelMA-HA solutions at higher concentrations can impede the diffusion of photoinitiators and radicals during UV exposure, thereby slowing down and reducing the efficiency of the crosslinking process, particularly in thicker samples. Additionally, the incorporation of HA can influence the gelation kinetics by creating a more hydrated and less densely crosslinked network. Conversely, when lower concentrations of HA (0.1–0.2%) are used, the overall bulk stiffness of the gelMA-HA network increases due to the reinforcement provided by HA through the formation of secondary bonds [77]. These results clearly demonstrate the role of HA in adjusting the overall mechanical properties of gelMA-HA hydrogel, providing an additional practical way to regulate the mechanical strength of gelMA-based hydrogel.

To further elucidate the mechanisms of mechanical alterations resulting from the addition of HA, SEM images were obtained, revealing the microstructures of gelMA-HA hydrogel substrates (Figure 2B–D). The overall porosity of the tested hydrogel samples ranged from 30% to 60%, with no clear trend observed when the HA concentration was varied from 0.1% to 1% compared to the pure GelMA sample. However, low HA concentrations (0.1–0.2%) led to a slight increase in average porosity, whereas higher concentrations (0.5–1%) resulted in a significant reduction in porosity (Figure 2B,C) [78]. The pore size distribution analysis indicated that the addition of HA increased pore size compared to pure gelMA samples, with higher HA concentrations (>0.5%) promoting the formation of significantly larger pores. This could explain the substantial reduction in bulk mechanical stiffness observed at high HA concentrations compared to pure gelMA or samples with lower HA content (Figure 2A,D). Taken together, the changes in pore size at the micro-level due to the addition of HA may elucidate the mechanisms underlying the changes in the mechanical properties of gelMA-based hydrogels. Since there was no clear trend in porosity variation, which is considered to be another major factor influencing the overall mechanical properties of hydrogels, we believe that the mechanical changes were likely due to the reinforcement of the sponge-like gelMA-HA matrix when a low concentration of HA was added.

**Figure 3:**
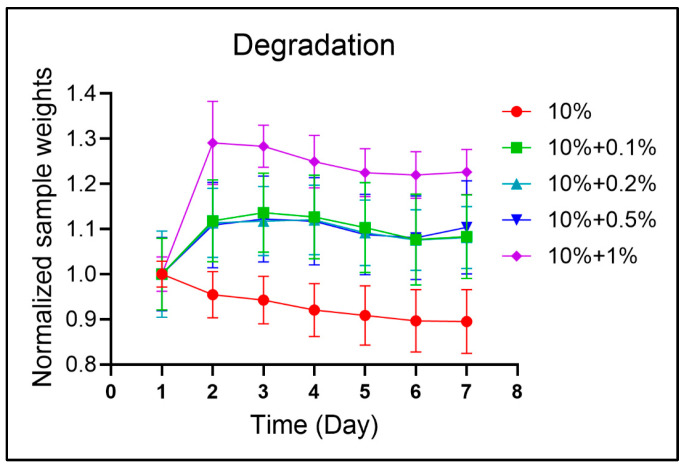
GelMA-HA hydrogel degradation analysis. Data for day 1 were normalized.

Hydrogels gradually degrade and soften over time. In GBM, invading tumor cells actively remodel the surrounding brain parenchyma by degrading the ECM [79], a process that directly influences cellular behavior and gene expression [80]. We evaluated the degradation behavior of gelMA-based hydrogels containing varying concentrations of HA (Figure 3). For 10% gelMA samples, degradation began immediately after gel formation, with approximately 10% mass loss observed after one week. The addition of HA altered the degradation profile. Specifically, HA-containing hydrogels exhibited an initial swelling phase within the first 24 h, followed by gradual degradation. When a high HA concentration (1% *w*/*v*) was incorporated, swelling increased significantly by up to 30% on day 1, indicating that HA substantially enhanced the water-absorbing capacity of the formed matrix. This behavior was attributed to the inherent hydrophilicity of uncrosslinked HA [81]. At higher concentrations, the unbound HA disrupted the gel network, increasing the mesh size and permitting greater water uptake and swelling. Overall, gelMA-HA hydrogels demonstrated a gradual degradation pattern over the first week, while the presence of HA markedly enhanced initial swelling behavior.

**Figure 4:**
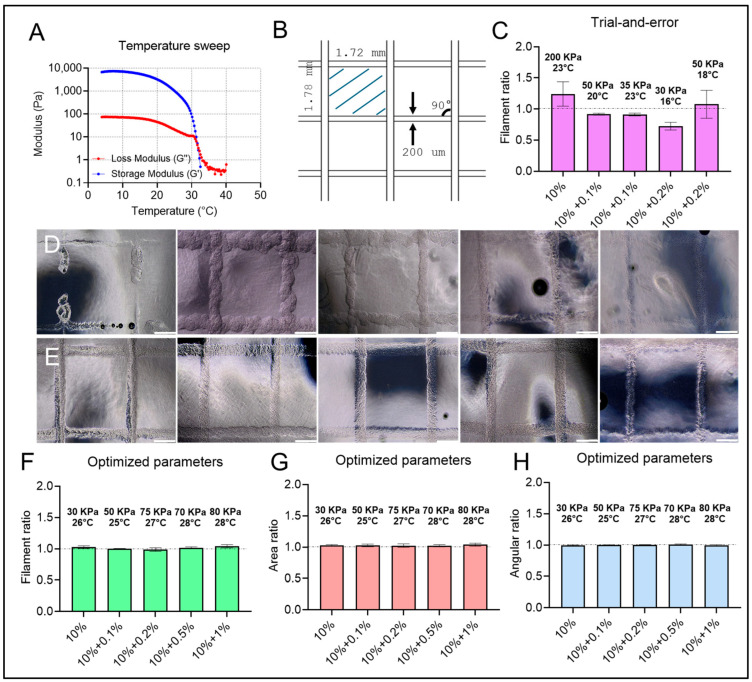
Optimization of the bioprinting process using a two-layer lattice model. (**A**) Rheological analysis through temperature sweep to determine the phase transition point of gelMA ink. (**B**) Schematic representation of the designed two-layer lattice model, including detailed geometric features. (**C**) Experimental trials evaluating suboptimal bioprinting parameters based on quantification of the printed model. (**D**) Bioprinted suboptimal models constructed using gelMA and gelMA-HA at varying concentrations. (**E**) Bioprinted models following process optimization. (**F**–**H**) Quantitative analysis of filament diameter, area, and angle, illustrating the effectiveness of the optimized bioprinting process. Scale bar: 500 µm.

In recent advancements, 3D bioprinting has emerged as a transformative tool in human disease modeling. It reshapes biomanufacturing by enabling the highly controlled fabrication of intricate, high-resolution constructs composed of ECM-like biomaterials and relevant cellular types, such as GBM cells [82]. However, maintaining high printing fidelity, particularly with soft biomaterials like hydrogels, poses a considerable challenge [83]. Common issues, including material spreading, fusion, and structural collapse, frequently compromise the structural integrity of printed constructs. This can ultimately diminish their effectiveness, misguide cellular regrowth, and potentially lead to complete failures in modeling human diseases. A proven approach to enhance the printability of hydrogel-based bioinks involves analyzing their flow behavior, specifically targeting viscoelastic properties. For optimal structural stability, bioinks with a storage modulus (G′) exceeding the loss modulus (G′′) are generally preferred, as a higher G′ offers mechanical elasticity that better supports layer-by-layer stacking of the material [60,84]. Given the thermo-sensitivity of gelMA-based hydrogel, we conducted a temperature sweep to define an appropriate temperature range for gelMA bioprinting. As illustrated in Figure 4A, both G′ and G′′ values declined as the temperature rose from 4 °C to 40 °C, with the two curves intersecting at 31 °C. This crossover confirmed the thermosensitive properties of the gelMA formulation, establishing 31 °C as the threshold where gelMA bioink transitioned from a predominantly elastic to a viscous state. Consequently, this temperature has been designated as the upper limit for our 3D bioprinting procedures, ensuring optimal construct stability.

Controlling the viscoelastic properties of hydrogel-based bioinks through temperature adjustments is a key advancement, yet challenges persist in achieving high structural accuracy when building 3D constructs such as human disease models. The material deformation, which is induced by gravity and the inherent spreading tendency of viscous bioinks, requires a refined approach. To address these challenges, the rigorous optimization of bioprinting parameters, including pressure and speed, is essential, alongside strategies to curb spreading post-deposition and support structural integrity. To achieve precise control over filament dimensions matching the inner geometry of printing nozzles, a mathematical model has been developed to harmonize bioprinting pressure and speed [85]. Besides bioprinting process modeling and optimization, recent advancements in embedded bioprinting have provided a new solution to deformation issues [86]. By embedding the bioink filaments within a shear-thinning hydrogel bath with a specific yield stress, this bath lifts the deposited filaments in place, stabilizes the constructs in material stacking, and, therefore, significantly enhances the overall structural accuracy. We prepared a 0.4% *w*/*v* Carbopol solution as the supporting bath, as identified in our previous research, for embedded bioprinting [58]. Through comprehensive experimentation, we optimized bioprinting conditions, including the bioprinting pressure, speed, and temperature, for fabricating gelMA-HA composite structures. This was specifically tested using a two-layer grid model to assess key metrics like filament diameter, grid area, and interlayer angles (Figure 4B). First, a consistent bioprinting speed of 5 mm/s was chosen to sustain both printing efficiency and smooth filament flow. Starting at a lower temperature range (16–23 °C), we encountered challenges with flow control, where higher pressures (>150 kPa) led to excessive bioink flow, while lower pressures (<30 kPa) interrupted flow continuity for all gelMA inks (Figure 4C). More importantly, maintaining a constant bioink flow rate in such low-temperature ranges required frequent adjustments to bioprinting pressure due to the thermosensitive properties of gelMA, complicating the process and, therefore, the printing fidelity (Figure 4D). By increasing the temperature incrementally from 24 °C to 30 °C, we observed enhanced bioink mobility, balanced with sufficient elasticity for stability within the support bath. Interestingly, the inclusion of HA required either a higher temperature or bioprinting pressure to achieve steady extrusion flow due to the inherent properties of HA that increased the overall bioink viscosity. To maintain low bioprinting pressure (below 100 kPa), minimizing the potential mechanical stress-induced cell damage when cells are encapsulated in the future [87,88], we prioritized temperature increases as a practical solution. This approach proved to be effective in achieving the consistent extrusion of gelMA-HA bioinks (Figure 4E). Following extensive experimental trials, we successfully identified optimal temperatures and pressures for various gelMA-HA formulations. This enabled the fabrication of constructs with features, including filament diameter, interlayer angles, and grid dimensions, closely matching the intended design within a 5% tolerance (Figure 4F–H).

**Figure 5:**
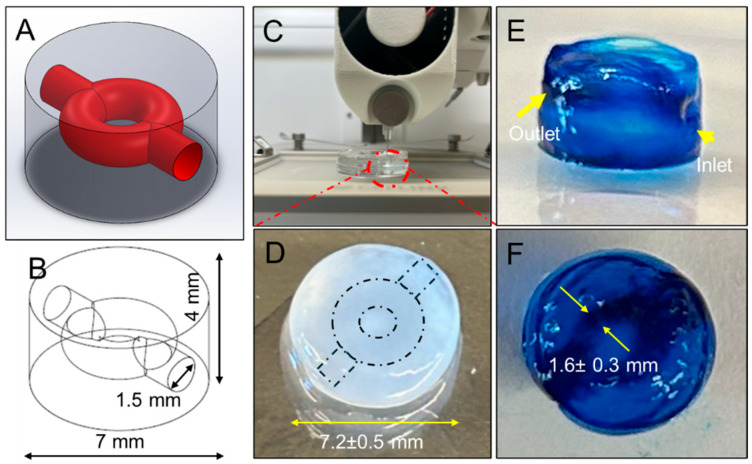
Design and bioprinting of an inner channel-incorporated 3D GBM model. (**A**,**B**), Schematic design of the GBM model with dimensional specifications. (**C**) Overview of the embedded bioprinting process utilized for model fabrication. (**D**–**F**), Bioprinted GBM model demonstrating high structural fidelity.

With the bioprinting processes rigorously optimized, we embarked on creating sophisticated 3D GBM models incorporating hollow channels using gelMA-HA bioinks (Figure 5A,B). A paramount challenge in developing vitro cancer models is the complete reproduction of the complex physiological landscape characteristic of tumors in vivo. This complexity arises from the convergence of multiple cellular and molecular components: not only tumor cells, but a myriad of stromal cells, signaling molecules, growth factors, and the ECM. Together, these elements form a dynamic, integrated TME, which fosters tumor growth, drives metastasis, and enhances resilience against apoptosis, even when subjected to aggressive therapies. Among the myriad of interactions within this intricate environment, the relationship between the tumor vasculature, GBM cells, and the ECM has emerged as profoundly influential, shaping essential GBM cell behaviors through demonstrated mechanisms such as angiogenesis and mechanotransduction [89,90]. Thus, constructing a 3D model that integrates ECM and vascular-like components is essential to reflect the physiological components of GBM. Our envisioned GBM model was crafted with these complexities, embodying a ring-shaped structure embedded with branched inner channels (Supplementary Video S1), which allowed the culture medium perfusion to mimic the dynamic conditions in GBM. In this design, the GBM cells encapsulated in the hydrogels could be deposited and positioned at the center. This spatial configuration was more than a structural feat, as it attempted to echo the intricate interactions of GBM cells within the TME, thus potentially offering a realistic platform for investigating the nuances of tumor progression, metastasis, and therapeutic resistance in dynamic perfusion. In this conceptual approval study, we successfully established 3D GBM constructs by leveraging the optimized bioprinting processes and the designed model, utilizing gelMA-HA composites (Figure 5C–F). These constructs exhibited high structural fidelity, with geometrical features that closely aligned with the original digital design. The overall and inner channel dimensions accurately reflected the intended geometries, with an acceptable margin of deviation. Notably, the ring-shaped inner channels maintained a fully open lumen, allowing seamless flow of dyed buffer through the structure. This fidelity in dimension and functionality underscores the robustness of our approach, enabling the precise modeling that is essential for capturing the dynamics of the TME in the future.

**Figure 6:**
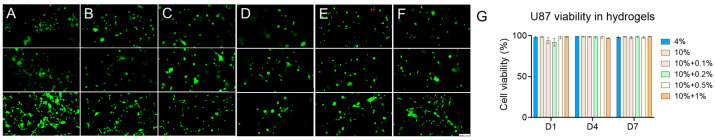
The viability of U87 encapsulated in hydrogel substrates. (**A**–**F**) Live/dead images (red color indicates dead cells, green color indicates live cells) obtained from different hydrogel substrates from 4% gelMA (**A**), 10% gelMA (**B**), and gelMA with HA from 0.1 to 1% (**C**–**F**) from day 1 to day 7. (**G**) Quantified cell viability. Scale bar: 200 µm.

**Figure 7:**
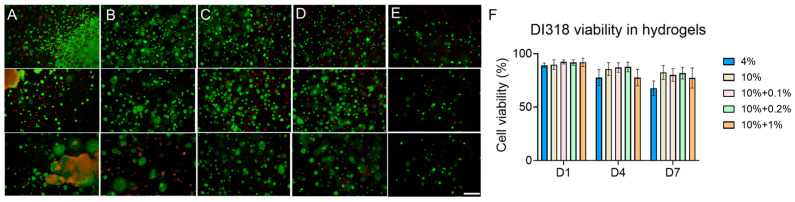
The viability of DI318 encapsulated in hydrogel substrates. (**A**–**E**) Live/dead images obtained from different hydrogel substrates from 4% gelMA (**A**), 10% gelMA (**B**), and gelMA with HA from 0.1 and 0.2 to 1% (**C**–**E**) from day 1 to day 7. (**F**) Quantified cell viability. Scale bar: 200 µm.

A fundamental requirement for 3D GBM modeling in vitro is ensuring robust cell viability, as high survival rates mirror in vivo conditions and therefore enable subsequent mechanistic studies. Cell viability within these models depends on a myriad of physical and chemical factors, particularly the mechanical properties and composition of the ECM. Research has consistently shown that GBM progression markedly increases the bulk stiffness of the TME (ranging from 0.1 to 47 kPa), contrasting sharply with the more compliant environment of normal brain tissue (0.1 to 2 kPa) [27,91,92]. This stiffening is largely attributed to the elevated expression of ECM components, including collagen and HA, secreted by GBM cells and stromal cells [93]. However, the specific influence of mechanical and compositional shifts on GBM cell viability and function within a 3D bioprinted ECM mimetic environment requires further exploration. Here, we conducted live/dead assays on GBM cells for both U87 and DI318 encapsulated within hydrogels featuring controlled stiffness and varying HA concentrations. The results showed that all tested bioinks sustained high cell viability, while the DI318 line showed more sensitivity to the hydrogel matrix with a higher ratio of dead cells compared to U87 and a tendency to form clusters, mimicking natural GBM morphologies (Figure 6 and Figure 7). This reflects the high turnover behavior characteristic of human GBM cells and highlights the potential limitations of using conventional cell lines in GBM research. The results also validate the biocompatibility of the gelMA-HA constructs. The 0.5% HA group was omitted in the DI318 culture, as prior results from U87 cells suggested that HA concentrations above 0.5% may negatively affect GBM cell behavior. Therefore, only the 1% HA group was included to represent a high HA concentration condition. These results are intriguing when considered alongside our mechanical stiffness data (Figure 2A) as they demonstrate that a bioprinted hydrogel model with stiffness exceeding the natural range of the GBM TME still supports high cell viability. These findings establish a solid foundation for employing these materials in 3D bioprinting to develop advanced GBM models for further mechanistic exploration.

**Figure 8:**
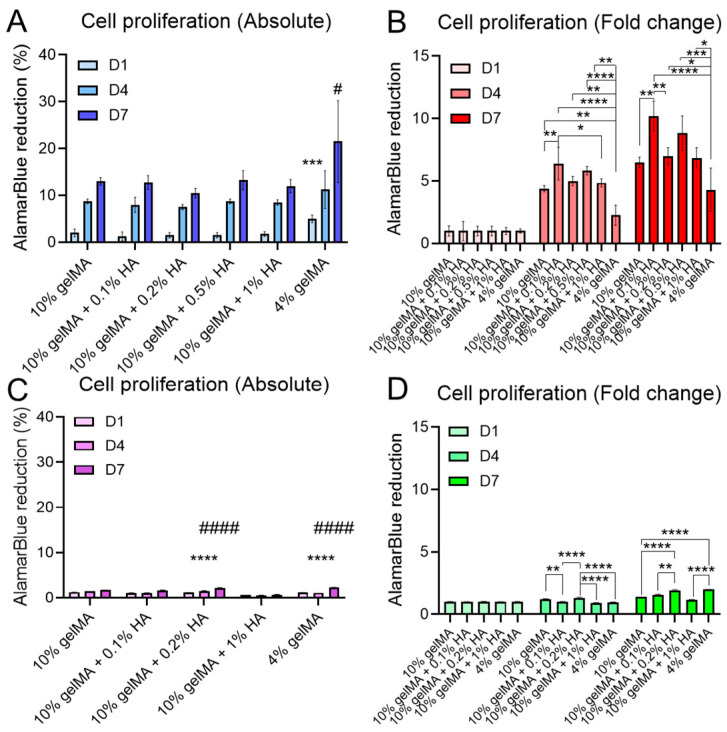
The proliferation of GBM cells evaluated using AlamarBlue assays. (**A**,**C**) Absolute AlamarBlue reductions in different gelMA and gelMA-HA groups for U87 (**A**) and DI318 (**C**). # is *p* < 0.05 and #### is *p* < 0.0001, indicating that the reduction for the 4% gelMA group was significantly higher than that of all other groups on day 7 for U87 and the reduction for the 4% gelMA and gelMA + 0.2% HA was higher than that for other groups on day 7 for DI318. *** is *p* < 0.001 and **** is *p* < 0.0001, indicating that the reduction for the 4% gelMA group was significantly higher than that for all other groups on day 4 for U87 and the reduction for the 4% gelMA and gelMA + 0.1% HA was higher than that for other groups on day 4 for DI318. (**B**,**D**) Fold-change in AlamarBlue reductions for all gelMA-HA groups for both U87 (**B**) and DI318 (**D**). *, **, ***, **** indicates *p* < 0.05 to *p* < 0.0001.

The relentless, uncontrolled growth of cells is a defining characteristic of tumors, making it important to consider the factors that influence and potentially regulate cell proliferation in GBM studies [12,94]. As evidence mounts, the TME has emerged as a crucial driver of GBM cell proliferation in vivo. Yet, the combined roles of mechanical stiffness and ECM components in modulating this proliferation are still not fully elucidated, requiring a more detailed investigation. To evaluate this further, we separately encapsulated U87 and DI318 within hydrogel-based models, seeking to replicate the inherent interactions between cells and the ECM observed in vivo. Through AlamarBlue assays, we explored how variations in ECM mechanical stiffness and the presence of HA influence the metabolic behavior of GBM cells within a 3D environment, reflecting their proliferating profiles. Our findings reveal that for U87, across all hydrogel groups—including pure gelMA at concentrations of 10% and 4% *w*/*v* and gelMA-HA composites with HA concentrations from 0.1% to 1% *w*/*v*—cells actively proliferate (Figure 8A,B). Notably, cells encapsulated in the 4% gelMA hydrogel (4.36 ± 1.15 kPa for 4% gelMA) exhibited significantly elevated AlamarBlue reduction values by day 1 compared to 10% gelMA and other groups, highlighting a pronounced initial proliferation in the softer ECM (Figure 8A). This observation aligns with previous studies, suggesting that in softer ECM environments, GBM cells experience fewer physical constraints, as these matrices possess less densely crosslinked polymer networks, which facilitate nutrient and oxygen infiltration [21]. In contrast, stiffer environments impose greater physical limitations, potentially curbing cell proliferation. Changes in ECM stiffness have also been linked to mechanotransduction pathways, which play an instrumental role in regulating cell behavior, including the proliferation of cancer cells, within a 3D matrix [95]. Upon normalizing the data using day 1 measurements as a reference, the proliferation fold-changes on days 4 and 7 for the 4% gelMA group showed a marked decline relative to the 10% gelMA and other groups (Figure 7B). This pattern suggests a growth trajectory within softer substrates, where rapid cell division occurs initially and then gradually decelerates as cells fill available space. Interestingly, when HA was added to the hydrogel matrix at varying concentrations, cell proliferation increased significantly, with the peak observed for 0.1% HA. In GBM tumors, excessive HA expression has been shown to promote proliferation, migration, and resistance to apoptosis, primarily through interactions with cell surface receptors like CD44 and RHAMM [96]. The presence of HA in our hydrogel substrate appeared to simulate a more authentic TME for GBM, fostering conditions conducive to cell replication. However, as HA concentration increased, a denser HA network likely formed within the hydrogel, which may have reversely affected the proliferation activity of encapsulated cells. It is noteworthy that the addition of a small amount of HA, which substantially enhanced the mechanical stiffness of the hydrogel substrate (Figure 2A), still led to a significant increase in cell proliferation compared to the pure 10% gelMA group. This finding highlights the predominant role of HA in promoting GBM cell proliferation compared to ECM mechanical stiffness. When DI318 cells were encapsulated, a significant reduction in proliferation was observed, as indicated by a much lower AlamarBlue reduction compared to U87 cells (Figure 8C,D). This is likely because U87 cells represent a more differentiated tumor phenotype characterized by high metabolic activity and rapid growth. In contrast, DI318 is a patient-derived, stem-like GBM cell line that typically proliferates more slowly and requires appropriate niche signals to support growth [97]. Nonetheless, DI318 cells exhibited similar proliferation trends as U87 over the course of one week. Notably, DI318 cells showed enhanced proliferation at low HA concentrations (0.1–0.2%), whereas proliferation declined when HA was high (1%). These results suggest that low HA concentrations support the proliferation of GBM cells. In the 4% gelMA samples, unlike U87 cells, DI318 cells exhibited a continuous increase in proliferation over one week. This trend is consistent with the U87 response to soft substrates, which are known to promote GBM cell proliferation and migration. However, due to the intrinsically slower proliferation rate, sufficient space remained available within the gel, leading to a sustained increase in AlamarBlue reduction for DI318. Given the significant differences between U87 and DI318 in terms of their viability and proliferation, and the more physiologically relevant characteristics of the DI318 cell line, we performed IHC exclusively on DI318 cells in the subsequent experiment.

**Figure 9:**
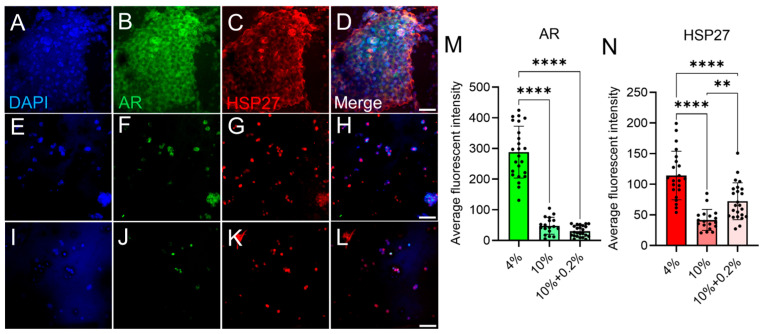
Immunostaining of DI318 cells encapsulated in hydrogel models. Cells in (**A**–**D**) 4% gelMA, (**E**–**H**), 10% gelMA, and (**I**–**L**), 10% gelMA + 0.2% HA. (**M**,**N**) Average fluorescent intensity of AR (**M**) and HSP27 (**N**) from different samples. ** is *p* < 0.005 and **** is *p* < 0.0001. Scale bar: 50 µm.

Despite decades of clinical efforts on GBM patients, current treatments offer mere palliation to patients with this devastating disease. Therefore, new targets for GBM treatment are imperative. It has been accepted that GBM involves a sex bias, impacting men more frequently [98]. Evidence has demonstrated that AR, the client protein of the HSP27, is frequently overexpressed in GBM and contributes to tumor progression [99,100,101]. However, how the mechanical properties and composition of the ECM impact AR and HSP27 expression in GBM cells has never been evaluated. Therefore, in this study, we aimed to investigate the relationship between ECM properties and GBM functional expression by evaluating AR and HSP27 levels using IHC. The results presented in Figure 9 align well with our proliferation findings, showing that GBM cells expressed significantly higher levels of AR and HSP27 in softer matrices (e.g., 4% gelMA), regardless of HA incorporation. Notably, larger cell clusters were frequently observed in the 4% gelMA group, with elevated AR and HSP27 expression localized near the periphery of clusters and diminished expression in their centers. In contrast, increasing the gelMA concentration to 10% led to a marked reduction in both AR and HSP27 expression (Figure 9M and N), along with less frequent cluster formation, indicating that stiffer matrices suppress GBM activity. Interestingly, the addition of 0.2% HA to the 10% gelMA matrix increased HSP27 expression compared to 10% gelMA alone. Given that HSP27 has been implicated in promoting apoptotic resistance in GBM under stress, this suggests a possible link between HA incorporation and enhanced resistance mechanisms, warranting further investigation into pathways through which HA may modulate GBM cell survival. Overall, our immunostaining data support the conclusion that matrix stiffness plays a dominant role in regulating GBM cell function, particularly AR and HSP27 expression, with softer environments promoting more biomimetic tumor phenotypes. Although our data indicate a role for HA in modulating GBM progression, we did not include matrices with constant low stiffness and varying HA concentrations. Thus, the specific contribution of HA, particularly under low mechanical constraint, remains to be fully elucidated. Please also note that we excluded the 0.1% and 1% HA groups from immunostaining analyses; based on our live/dead and proliferation data, we determined that the 0.2% HA group was the most representative, while the 1% HA condition appeared unfavorable for human GBM cells.

Based on these findings, gelMA-based hydrogels demonstrate strong potential as a platform for 3D GBM modeling. These hydrogels not only support the sustained survival and proliferation of GBM cells in vitro for at least seven days but also allow for seamless integration with ECM components, such as HA, enabling mechanistic investigations into how ECM properties influence GBM behavior. The ability to precisely control these component ratios enables a nuanced exploration of the physical and chemical cues in the TME that influence GBM progression. Further enhanced by embedded bioprinting techniques, the development of GBM models offers high reproducibility and refined geometric features. This allows not only for the incorporation of ECM materials but also the strategic placement of other critical components, such as dynamic flow and stromal cell types, into predesigned locations to mimic the architecture of natural tumors. The conceptual validation of this approach thus lays robust groundwork for in-depth mechanistic studies at the cellular and molecular levels, potentially propelling advancements in understanding GBM biology and accelerating therapeutic development. It should be noted that, while this study successfully established inner vascular channels within the model with high structural fidelity, we did not conduct dynamic culture experiments to assess functional parameters such as vascularization, permeability, or molecular diffusion using multiple cell types. The primary objective of this work was to demonstrate that the bioprinting process can be optimized to fabricate complex architectural features and validate their potential application in GBM modeling. Building on the achievements of this study, future work will incorporate multicellular cultures—including endothelial cells, astrocytes, and pericytes—under dynamic culture conditions to enable a more comprehensive analysis of GBM progression and evaluate potential chemotherapeutic strategies targeting the AR-HSP27 axis.

## 4. Conclusions

This research presents an advanced yet practical 3D bioprinting approach for creating in vitro GBM models with tunable physical and chemical properties and high structural fidelity. These models, constructed from naturally derived hydrogels, serve as versatile platforms to investigate key contributors to the GBM TME, such as the influence of ECM properties on GBM cell functions. Our findings demonstrate that the microporous structures of the hydrogel substrate can be precisely modulated by adjusting the concentrations of gelMA and HA. Higher HA concentrations can increase pore size, although no clear trend in overall porosity was observed. These microstructural changes are directly linked to the bulk mechanical behavior and swelling of the hydrogels, with larger pore sizes resulting in decreased mechanical strength but enhanced swelling ratio. Rheological analysis identified a range in which the G’ remained higher than the G’’, a criterion widely accepted for good printability. Based on this analysis, bioprinting parameters, including temperature and printing pressure, were systematically optimized via trial and error. Using these parameters, complex 3D GBM models incorporating intricate internal channels were successfully bioprinted with high structural accuracy. Biocompatibility testing confirmed the cytocompatibility of the hydrogels. However, proliferation results varied with the tuning of gelMA and HA combinations. Low-stiffness hydrogels promoted increased cell proliferation for both U87 and human DI318 cell lines, while the addition of HA at relatively low concentrations enhanced the proliferation rate. GBM cells exhibited higher expression of AR and HSP27 in softer matrices, whereas the addition of low HA concentration enhanced HSP27 expression under stiffer matrix conditions. Building upon this established model, future studies can investigate the influence of ECM properties and flow dynamics on GBM progression, incorporating multiple cell types such as endothelial cells and astrocytes. These advancements may enable the development of more physiologically relevant tumor models, highlighting the potential of this platform for applications in personalized medicine.

## Data Availability

The data that support the findings of this study are available from the corresponding author upon reasonable request.

## Supplementary Materials

The following supporting information can be downloaded at https://www.mdpi.com/article/10.3390/mi16060654/s1: Video S1: channeltesting7.23.24.mov.

## Abbreviations

The following abbreviations are used in this manuscript:

MDPI	Multidisciplinary Digital Publishing Institute
DOAJ	Directory of open access journals
GBM	Glioblastoma
TME	Tumor microenvironment
ECM	Extracellular matrix
HA	Hyaluronic acid
GelMA	Gelatin methacryloyl
